# Effect of Phage Spray on Hatchability and Chick Quality of Eggs Contaminated with *Salmonella* Typhimurium

**DOI:** 10.3390/v16081338

**Published:** 2024-08-21

**Authors:** Leping Wang, Qinting Dong, Kunping Tang, Kaiou Han, Huili Bai, Yangyan Yin, Changting Li, Chunxia Ma, Ling Teng, Jun Li, Yu Gong, Yuying Liao, Hao Peng, Xiaoye Wang

**Affiliations:** 1 Guangxi Key Laboratory of Animal Reproduction, Breeding and Disease Control, Guangxi Zhuang Autonomous Region Engineering Research Center of Veterinary Biologics, College of Animal Science and Technology, Guangxi University, Nanning 530003, China; wangleping96@foxmail.com (L.W.); tangkunping0424@foxmail.com (K.T.); huilibai2020@126.com (H.B.); machunxia1213@163.com (C.M.); 2 Guangxi Key Laboratory of Veterinary Biotechnology, Guangxi Veterinary Research Institute, Nanning 530001, China; yinyangyan@163.com (Y.Y.); lctyq0508@163.com (C.L.); 15208987486@163.com (L.T.); jlee9981@163.com (J.L.); 3 Guangxi Vocational University of Agriculture, Nanning 530009, China; dongqinting@foxmail.com; 4 Animal Disease Prevention and Control Center, Guilin 541000, China; hankaiou@sina.com; 5 Animal Science and Technology Station of Guizhou, Guiyang 550018, China; yituo-28@163.com

**Keywords:** phage, egg hatching, disinfection, *Salmonella* Typhimurium

## Abstract

*Salmonella* Typhimurium (*S.* Typhimurium) contamination poses a significant challenge to breeder egg hatchability and chick health, necessitating the exploration of alternative disinfection methods. This study investigates the potential of phage vB_SPuM_SP02 (SP02) as a novel disinfectant for breeder eggs contaminated with *S.* Typhimurium SM022. Phage SP02 was isolated from poultry farm effluent and characterized for morphology, biological properties, and genome properties. Experimental groups of specific pathogen-free (SPF) eggs were treated with *Salmonella* and phage SP02, and efficacy was assessed through hatching rates, chick survival, weight, *Salmonella* load, immune organ indices, and intestinal flora. Phage treatment effectively eradicated *Salmonella* contamination on eggshells within 12 h, resulting in increased hatching and survival rates compared to controls. Furthermore, phage treatment mitigated weight loss and tissue *Salmonella* load in chicks without causing immune organ damage while reducing *Salmonella* spp. abundance in the intestinal tract. This study demonstrates the potential of phage SP02 as an eco-friendly and efficient disinfectant for *S*. Typhimurium-contaminated breeder eggs, offering promising prospects for practical application in poultry production.

## 1. Introduction

*Salmonella* stands as a significant pathogen with profound implications for the poultry industry, curtailing economic gains. Its entry into poultry flocks can occur through various channels, including direct transmission between flocks, contaminated feeds, biological vectors (such as insects, rodents, wild birds, and humans), and vertical transmission [1]. *Salmonella* Typhimurium (*S.* Typhimurium) is one of the important *Salmonella* serotypes associated with eggs [2]. The factors that are responsible for its epidemic spread still remain unclear. Epidemiological investigations indicate that laying flocks become infected directly from the farm environment [2,3]. It is also known that egg contamination by *S*. Typhimurium may occur by vertical transmission in the reproductive tract before deposition of the shell [4]. Excessive contamination of these eggs can lead to a decrease in incubation capacity, quality, growth, and performance of the chicks [5]. However, the competitiveness of modern intensive poultry production creates the need to achieve high efficiency and to optimize hatchability, as well as chick viability and growth performance [6]. All of these are fundamentally dependent on the disinfection of hatching eggs [7].

Thus, using an effective sanitizer on the eggshell surface is important for reducing the potential for external and internal contamination. However, the inadequate application of sanitizers allows microorganisms to penetrate the eggshell pores and reach the embryo [8]. These microorganisms disrupt the embryo and consequently reduce the incubation efficiency [9]. Traditional measures to prevent and control *S.* Typhimurium-contaminated eggs include the use of ormaldehyde fumigation, but this treatment has toxic effects, causing embryonic deaths and a deterioration in chick quality [10,11]. Other authors [12,13] have reported that formaldehyde fumigation should be excluded from decontamination programs for breeding eggs, despite its efficacy as a disinfectant, due to exposure of farm workers or hatchery personnel to the toxic and potentially carcinogenic compound [14]. Some hatcheries use disinfectants for breeding eggs that are safe for human health and the environment. For this purpose, ozone, hydrogen peroxide, ethyl alcohol, or chlorine dioxide substances have been used in previous experiments as an alternative to formaldehyde for the disinfection of eggs, incubators, and livestock houses. These also have problems. Sander and Wilson showed that H_2_O_2_ application for egg disinfection resulted in a significant loss of moisture (egg mass) during incubation and limited the number of absorbed yolk sac in 42-day-old broilers [15]. One study proved that the application rate of ClO_2_ evaluated was not an effective antimicrobial alternative to formaldehyde for sanitizing breeding eggs in hatcher cabinets prior to hatch [16]. Another finding indicated that ozone is a good disinfectant yet may adversely affect embryo development when given in a gaseous form [17]. Therefore, there is an urgent need to develop new methods of egg disinfection that are highly bactericidal and harmless to breeding embryos.

Phage biocontrol is increasingly accepted as a natural and green technology; it has advantages of easy availability, specificity, harmlessness to the public, and being environmentally friendly compared to conventional antimicrobials. Most research into phages as antibacterial agents has focused on applications such as using phages to treat bacterial infections in patients due to antibiotic-resistant bacteria [18,19]. Less attention has been paid to the opportunity to use phages as biocontrol agents in the environment [20]. Yet, increasingly, phages are being used in other industries, such as for medical devices [21], agriculture [22], aquaculture [23,24], and the food industry [25], as prophylactic treatments to prevent disease. However, challenges remain in the widespread implementation of phage treatments, including the development of phage resistance, the regulation of phage-based products, and the unknown environmental consequences of phage treatment [26]. While there are many challenges, there are also numerous benefits of phage treatment, including better control and penetration of biofilms [27], the re-sensitization of antibiotic-resistant bacteria [28], and the ability to evolve to evade bacterial defenses [29].

However, there are few reports on the application of phages for egg incubation disinfection. The objective of this study was to assess the ability of bacteriophage administration by conducting an experimental model with phage vB_SPuM_SP02 (designated as SP02) and *S*. Typhimurium SM022 contaminating specific pathogen-free (SPF) eggs to investigate the feasibility of phages as a novel disinfectant for breeder eggs.

## 2. Results

### 2.1. Analysis of Morphology and Characteristics of Phage SP02

**Figure 1:**
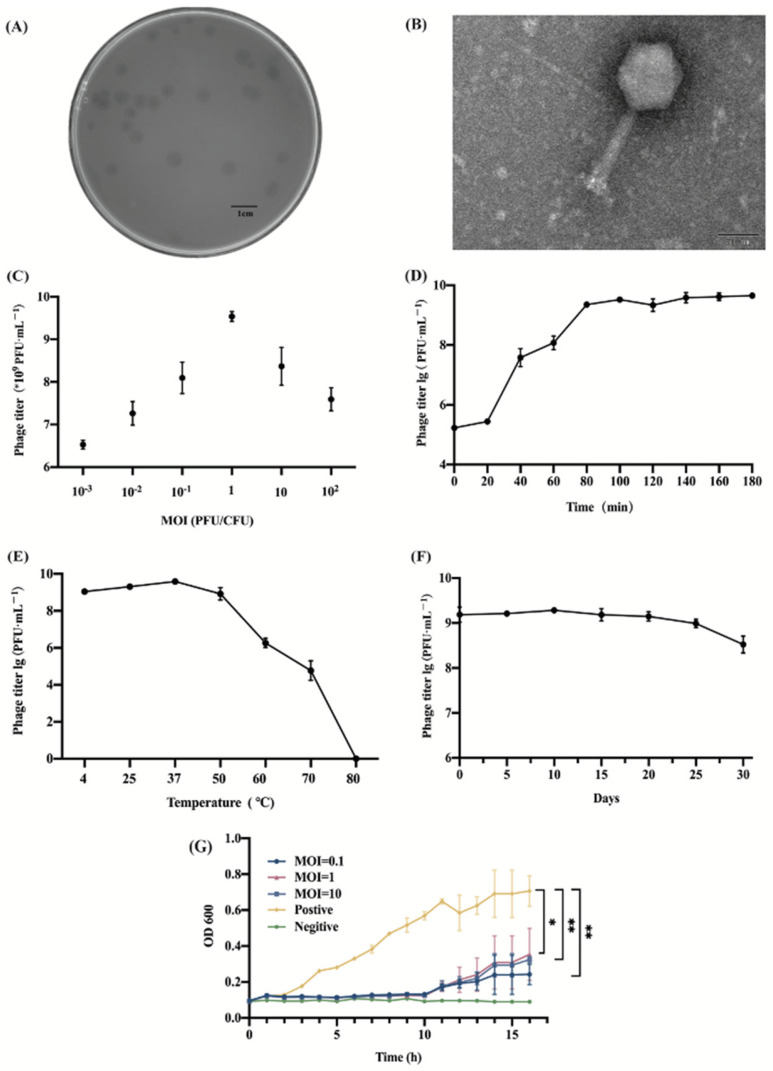
Morphology and biological characterization of phage SP02. (**A**) Phage plaque of phage SP02; (**B**) TEM of phage SP02; (**C**) optimal multiplicity of infection; (**D**) one-step growth curve; (**E**) stability at different temperatures from 4 to ~80 °C· for 1 h; (**F**) stability at 37 °C for one month; and (**G**) in vitro sterilization profiles of phage SP02 at different MOI. “*” Bars with different signs differ significantly (*p* < 0.05), “**” Bars with different signs differ extremely significantly (*p* < 0.01).

A phage was isolated from avian farm sewage by using *S*. Typhimurium SM022 as the host strain and named vB_SPuM_SP02 (designated as SP02). The plaque morphology observed on plates showed that SP02 was able to form a clear and bright plaque using SM022 (Figure 1A). Furthermore, the plaque was 3 mm in diameter (Figure 1A). TEM analysis showed that SP02 had an icosahedral shape with a stereo-symmetric head and a long, contractile tail. The head was a typical regular polyhedral shape with a diameter of 45 ± 2 nm. The tail length and diameter were approximately 150 ± 2 nm and 10 ± 0.5 nm, respectively (Figure 1B).

When the multiplicity of infection (MOI) was one, the phage titer was the highest (Figure 1C). Three groups of parallel experiments were conducted for each MOI. It can be seen the incubation period of the phage was about 20 min, the lysis period was about 100 min, and the burst size of the phage was about 134/cell (Figure 1D).

In the temperature stability test, the phage titer of the phage hardly changed at 4–50 °C. When the temperature was higher than 50 °C, the phage titer started to decrease, and when it reached 80 °C, the phage was completely inactivated (Figure 1E). At 37 °C, the phage titer started to decrease on day 20, and on day 30, the phage titer decreased by 0.75 orders of magnitude compared to the initial phage titer (Figure 1F).

The bactericidal efficacy of the phage in the medium was assessed by measuring the OD_600_ value (OD, optical density) by co-culturing the phage SP02 with the host bacterium SM022. The positive group had only host bacteria without the phage, and the OD_600_ values tended to increase and remained at a high level during the 16 h incubation. Within 10 h, all three groups of MOI could inhibit bacterial growth. After 10 h of incubation, the phage could not inhibit the growth and reproduction of bacteria, and the bacterial concentration gradually increased. The difference in the inhibition effect of the three groups of MOI was not obvious. The best inhibition effect was achieved when the MOI = 1, which was also consistent with the best MOI (Figure 1G).

The results of phage SP02 host range identification are shown in Table 1, The phage could lyse two strains of *S.* Typhimurium (CVCC3384, SM022), two strains of *S*. Pullorum (SF-0923, SX-1014), and one strain of *Pseudomonas aeruginosa* (SZPA-PA1).

### 2.2. Whole Genome Sequencing Analysis of Phage SP02

**Figure 2:**
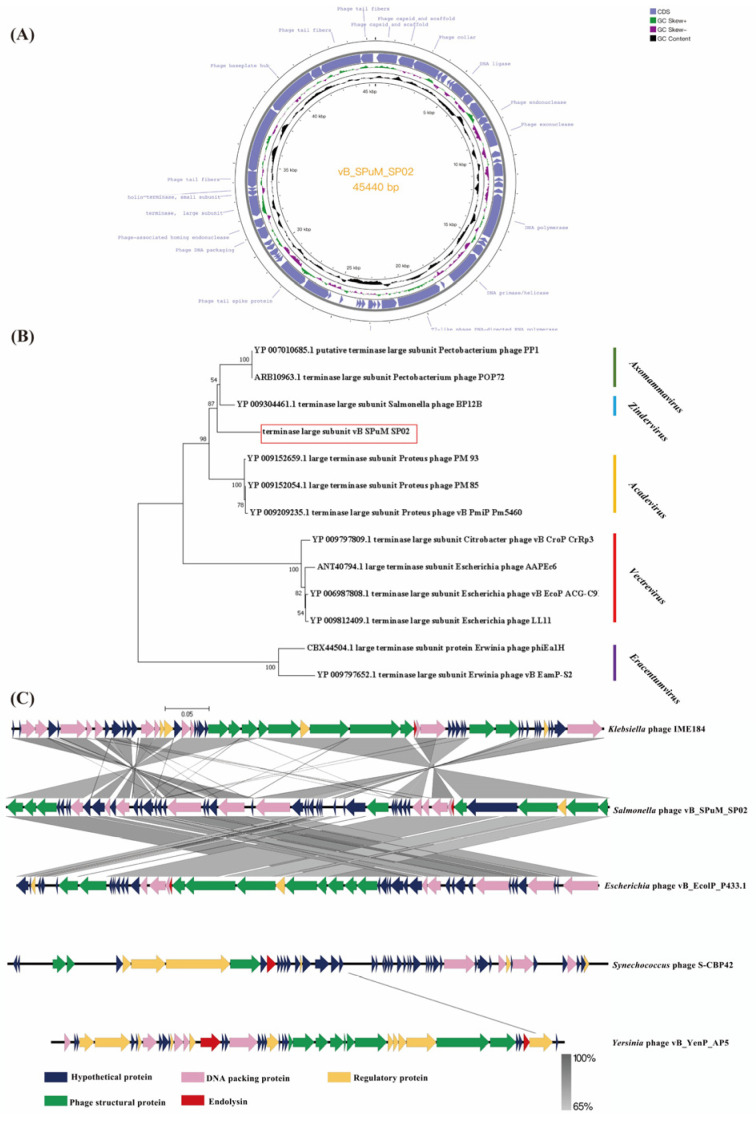
Phage SP02 whole genome analysis. (**A**) Genome map of phage SP02; the genome of phage SP02 was depicted as circular. The arrows represent 54 ORFs, among which 20 ORFs were predicted to have known function. In addition, the map shows GC skew and content about the genome. Note: unmarked ORFs in the graph are hypothetical proteins; (**B**) Phylogenetic analysis based on the sequences of large terminase subunit proteins from 13 phages using MEGA 7 with 1000 bootstraps. Red frame locates the phage SP02. All phages are from different genera of the subfamily *Molineuxvirinae*; (**C**) Comparative analysis of the whole genome of five phages. Blue arrows indicate hypothetical protein, other colored arrows indicate conserved proteins with homology.

The genome of bacteriophage SP02 was 45,440 bp in length, and the GC content was 47% (Figure 2A). BLASTn analysis showed that the whole genome of SP02 had an identity of 78.52% with *Klebsiella* phage IME184 (GenBank: MZ398244); RAST online annotation results indicated that the genome contains 54 open reading frames (ORFs), of which 20 ORFs (37.04%) were predicted to encode functional proteins. Genes associated with virulence, toxins, and antibiotic resistance factors were absent in the genome, suggesting the safety of SP02 being used in phage therapy.

Genomic BLASTn nucleic acid results showed that the bacteriophage genome has low homology with the bacteriophage genomes currently published on National Center for Biotechnology Information (NCBI). According to BLASTn, the phage belonged to the family *Molineuxvirinae*. The phage SP02 genome was compared with that of other phages in the family *Autographiviridae.* Among the phages, phage SP02 was the most homologous to the *Klebsiella* phage IME184 (MZ398244.1). *Synechococcus* phage S-CBP42 (KC310805.1), *Yersinia* phage vB_YenP_AP5 (KM253764.1), and *Ralstonia* phage RPSC1 (MF893341.1) shared no homology with phage SP02 (Figure 2C). This indicated that phage SP02 may be a new member of the subfamily *Molineuxvirinae*.

In order to understand the evolutionary relationship between this bacteriophage and another subfamily, *Molineuxvirinae*, we chose the relatively conserved phage terminal large subunit (ORF46) gene adopting the adjacency method in MAGE 7 software (Ver:7.0.26) to build a phylogenetic tree. The results showed that phage SP02 was located at the up branch with the genus *Zindervirus*, and down branch with the genus *Axomammvirus* phages. Phage SP02 is a unique branch in the subfamily *Molineuxvirinae* (Figure 2B). Our results suggested that phage SP02 may belong to one of the new genera in the class *Caudoviricetes*, family *Autographiviridae*, and subfamily *Molineuxvirinae*.

### 2.3. Phage Protective Effect on Hatchability of Egg Infected with S. Typhimurium

**Figure 3:**
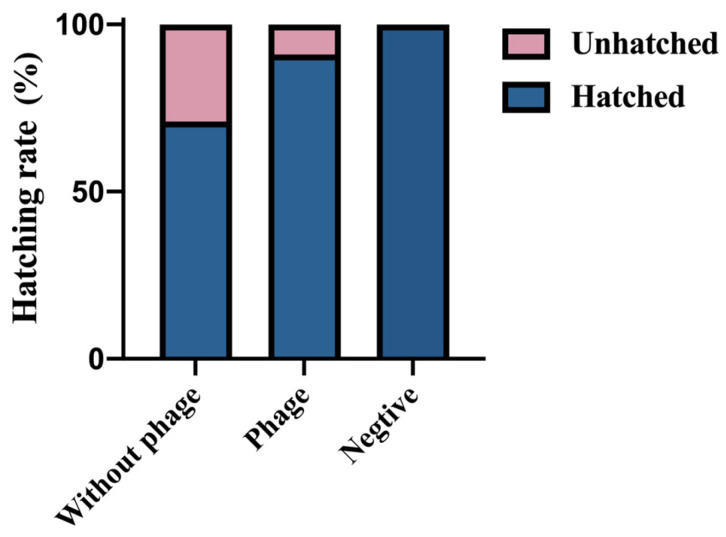
Hatching rate of chicks after phage SP02 disinfection.

All breeding eggs were checked for normal development by day 18 of incubation. On day 21, chicks emerged from the shell, and the hatching rate of breeding eggs was 100% in the negative group, 91.11% in the phage group, and 71.11% in the without phage group (Figure 3).

### 2.4. Phage Protective Effect on Chicks Two Weeks after Hatching Infected with S. Typhimurium

**Figure 4:**
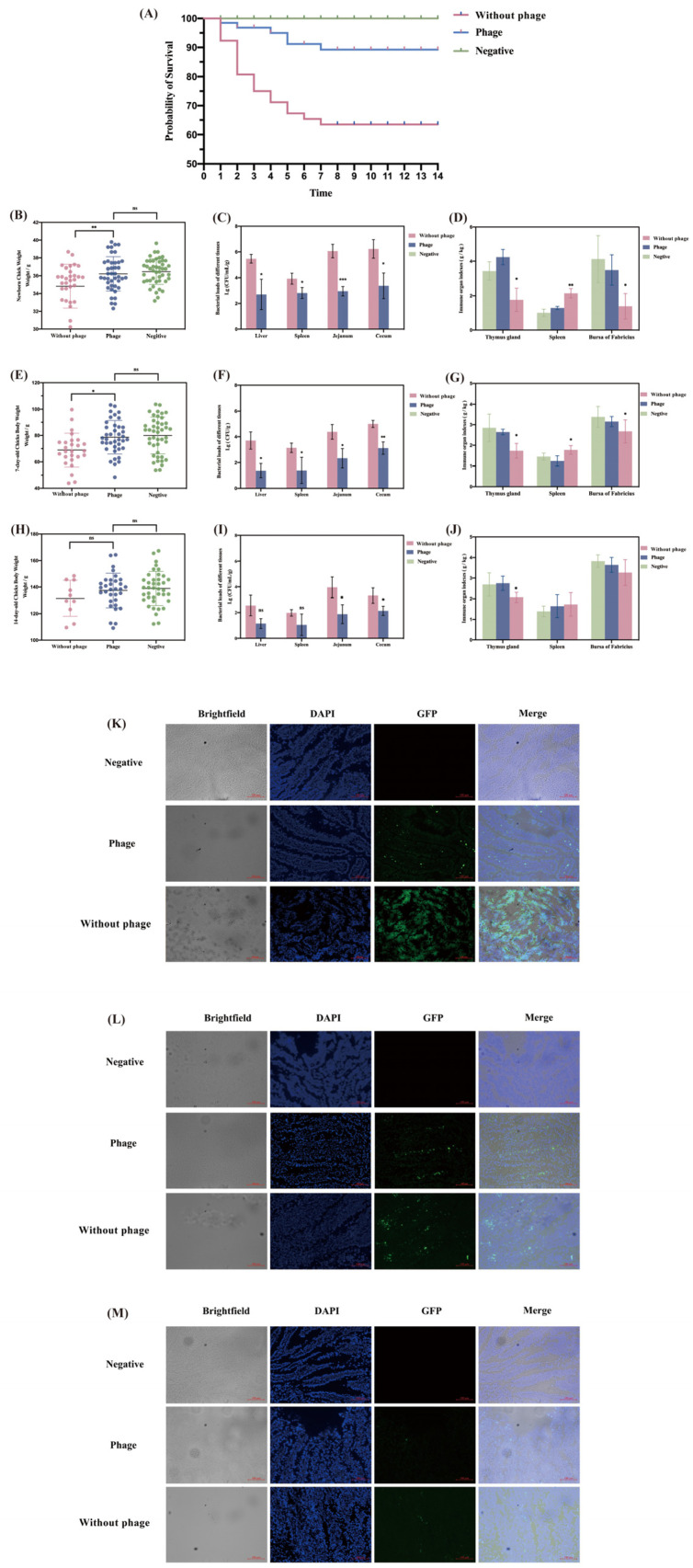
Changes in chicks after hatching of breeding eggs. (**A**) Hatching rate of chicks after phage SP02 disinfection; (**B**) changes in chick weight on day 2; (**C**) *Salmonella* load in chick organs on day 2; (**D**) immune organ index of chicks on day 2; (**E**) changes in chick weight on day 7 after hatching; (**F**) *Salmonella* load in chick organs on day 7 after hatching; (**G**) immune organ index of chicks on day 7; (**H**) *Salmonella* load in chick organs on day 14 after hatching; (**I**) changes in chick weight on day 14 after hatching; (**J**) immune organ index of chicks on day 14; (**K**) observation of frozen sections of chick jejunum on day 2 (200×); (**L**) observation of frozen sections of chick jejunum on day 7 (200×); and (**M**) observation of frozen sections of chick jejunum on day 14 (200×). “*” Bars with different signs differ significantly (*p* < 0.05), “**” Bars with different signs differ extremely significantly (*p* < 0.01), “***” Bars with different signs differ ultra-significantly (*p* < 0.001).

When observing the survival of chicks in three groups for 14 days, the survival rate was 89.25% in the phage group, 63.46% in the positive group, and all chicks in the negative group survived (Figure 4A).

According to the results (Figure 4B–D), the average weight of chicks in the without phage group was 34.55 g and that in the phage group was 36.20 g. For the without phage group, the chick’s weight was significantly lower than the phage group on the first day (*p* < 0.01). On the seventh day, the average weight of chicks in the without phage group was 68.94 g and that in the phage group was 78.60 g. For the without phage group, the chick’s weight was significantly lower than the phage group (*p* < 0.05). On the fourteenth day, the average weight of chicks in the without phage group was still lower than that in the phage group, but there was no difference in the statistical analysis (*p* > 0.05).

Bacterial loads were found in the liver, spleen, jejunum, and cecum of day 1, 7, and 14 chicks (Figure 4E–G). Compared with the without phage group, the bacterial load was reduced in all organs at different times in the phage group. And in the without phage and phage group, the bacterial load also decreased gradually at 1, 7, and 14 days. We observed the inter-tissue fluorescence expression using fluorescence microscopy after freezing sections of jejunum at 1, 7, and 14 days, which was also consistent with the bacterial load results (Figure 4H–J).

As shown in the results (Figure 4K–M), there was a significant decrease in the thymus index of chicks in the without phage group compared to the phage group on days 1–14 (*p* < 0.05). There was a significant increase in the spleen index of chicks in the without phage group compared to the with phage group on days 1–7 (Day 1 *p* < 0.01; Day 7 *p* < 0.05), although the increasing trend gradually slowed down and there was no statistical difference on day 14 (*p* > 0.05). There was a significant decrease in the fasciola bursa index in the phage-free group compared to the phage group on days 1–7 (*p* < 0.05), with no statistical difference on day 14 (*p* > 0.05).

### 2.5. Changes in Abundance of S. Typhimurium in Intestinal Tract of Hatching Chicks

**Figure 5:**
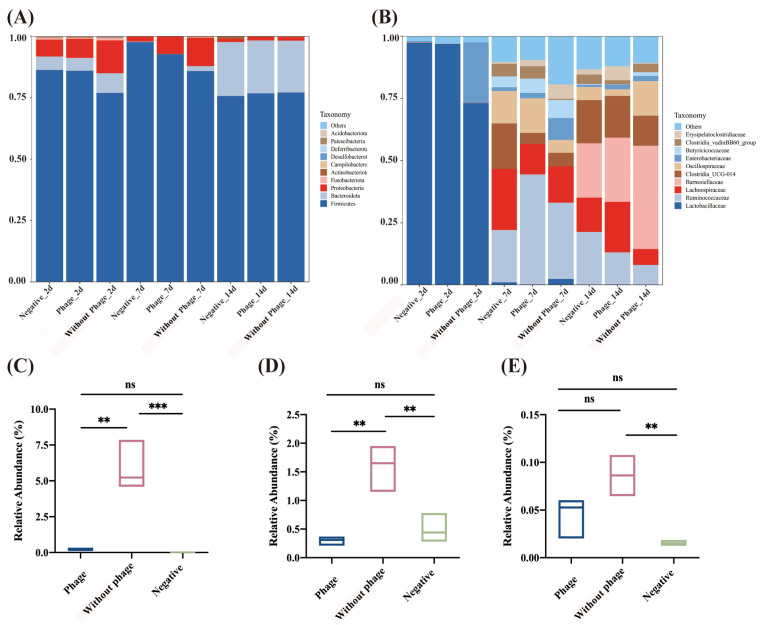
Microflora structure of the chick cecum at the level of the phylum and the family. (**A**) Microflora structure of the chick cecum at the phylum level; (**B**) microflora structure of the chick cecum at the family level; (**C**) differential species abundance at the level of *Salmonella* spp. in chicks on day 2; (**D**) differential species abundance at the level of *Salmonella* spp. in chicks on day 7; and (**E**) differential species abundance at the level of *Salmonella* spp. in chicks on day 14. “**” Bars with different signs differ ex-tremely significantly (*p* < 0.01), “***” Bars with different signs differ ultra-significantly (*p* < 0.001).

Detection and analysis of the top 10 phylum and family compositions of the gut microbiota were determined for all groups. The gut microbiota is predominantly composed of several key phyla, which include *Firmicutes*, *Bacteroidota*, *Proteobacteria*, *Actinobacteriota*, *Fusobacteriota*, *Campylobacterota*, *Desulfobacterota*, *Deferribacterota*, *Patescibacteria*, and *Cyanobacteria* (Figure 5A). The dominant families in the cecal content are *Lactobacillaceae*, *Ruminococcaceae*, *Barnesiellaceae*, *Clostridia*, *Oscillospiraceae*, *Enterobacteriaceae*, *Enterococcaceae*, *Eubacterium*, *Erysipelotrichaceae*, and *Butyricicoccaceae* (Figure 5B). As shown, comparisons revealed that the phage group reduced Proteobacteria by 5.68%, 4.45%, and 0.04% on days 2, 7, and 14, respectively, compared to the positive control group. *Enterobacteriaceae* decreased by 24.00%, 6.62%, and 0.25% on days 2, 7, and 14, respectively.

To further compare the changes in *Salmonella* abundance at the genus level within the gut microbiota, it was observed that on the 2nd and 7th days, there was a significant decrease (*p* < 0.01) in *Salmonella* relative abundance in the phage group compared to the without phage group. However, on the 14th day, the difference in relative abundance was not significant (*p* > 0.05) (Figure 5C–E).

## 3. Discussion

Currently, the most widely used method for disinfecting eggs during the incubation process in the hatchery industry is the use of chemical disinfectants such as formaldehyde [30]. However, studies have indicated that residual formaldehyde gas after fumigation can have adverse effects on the health of workers in poultry farms [31,32]. Formaldehyde is known to cause strong irritation to the eyes and nose, with risks of diseases such as nasopharyngeal cancer and leukemia. Consequently, in 2014, the European Union officially reclassified formaldehyde as a carcinogenic, mutagenic, and acutely toxic compound, prohibiting its use in the incubation process of eggs. In Germany, gaseous formaldehyde is restricted to certain industries only [33]. While there is no explicit regulation in China prohibiting the use of formaldehyde for disinfecting eggs during incubation, there is a growing trend toward developing new disinfectants for eggs and finding alternatives to formaldehyde. A strain of *Salmonella* phage SP02 was isolated from sewage. In addition, it was found to be stable at 37 °C for more than 30 days, and possessed sufficient antibacterial effects in vitro. Therefore, we believe that SP02 has good potential for application in the disinfection of eggs in incubators.

Hatch rate represents the effect of *S*. Typhimurium SM022 on unshelled chicks. In this study, the results showed that the hatching rate was higher in the phage group than in the without phage group. *S*. Typhimurium could penetrate eggshells and cause the death of breeding eggs [34]. We also isolated fluorescently labeled *S*. Typhimurium SM022 from dead breeding eggs, which also supports this opinion. Meanwhile, another study found that spraying phage on the surface of eggshells contaminated with *Salmonella* resulted in a significant decrease of 2.9 logs of *Salmonella* on the eggshell surface after 6 h and already exceeded the detection level after 24 h [35]. Therefore, we concluded that spraying phage SP02 on the eggshell reduced the content of *S.* Typhimurium, resulting in a higher hatching rate in the phage group than in the without phage group. However, the phage group also showed chicken embryo death and detected *S.* Typhimurium containing green fluorescence, probably due to the high concentration of *S.* Typhimurium preventing the phage from completely removing the *Salmonella* from the eggshell surface.

We tracked the health of chicks after hatching and observed their health from a survival rate perspective. Furthermore, many studies have shown that the use of phages for less than a week can lead to the disappearance of pathogens in chicks. A similar study found that after infection with *Salmonella*, phage treatment significantly increased the weight loss of chicks and substantially reduced the liver/body and spleen/body weight ratios, organ bacterial loads, the degree of hepatic sinusoidal dilatation, and congestion [36]. In another study by Waseh et al., 2-day-old pathogen-free chicks were used with a phage after 1 h of inoculation with 10^7^ CFU/mL *Salmonella*. The number of *Salmonella* decreased 100-fold over the next four days [37]. Bardina et al. studied phage potency over time, and the results showed that phage count does not decrease significantly over time after a few days of intestinal infection with *S.* Typhimurium, while the number of *S.* Typhimurium was reduced to 10^5^ CFU/mL after 12 days compared to the control group [38]. This is also consistent with our findings; chicks stopped dying 7 days after fledging, and compared to the without phage group, the phage group had significantly reduced weight loss, substantially reduced intra-organ *S*. Typhimurium levels, and improved immune organ indices. In addition, by the 14th day, there were no significant differences in the weight, *Salmonella* count, and immune organ indices between the phage group and the without phage group. We conjecture this may be due to the death of weak chicks or an increased resistance to *Salmonella* in the chicks at this time.

The thymus, spleen, and bursa of Fabricius are crucial immune organs in poultry growth and development [39,40]. Changes in their organ indices are important indicators of immune function. The bursa of Fabricius supports the differentiation and maturation of B cells, while the spleen, containing both B cells and macrophages, plays a vital role in humoral immunity [41]. The thymus is responsible for producing and maturing T lymphocytes and B lymphocytes, both of which are key immune cells in the immune system [42]. Research has shown that infection with *Salmonella* can lead to decreased thymus weight and atrophy, as well as acute atrophy of the bursa of Fabricius and disruption of its follicles [43]. Additionally, *Salmonella* infection can significantly increase the spleen coefficient in chicks [36]. In this study, similar results were observed, where chicks in the positive control group showed significant decreases in thymus and bursa of Fabricius indices on days 2 and 7, along with a significant increase in the spleen index compared to the phage group. This suggests that phage treatment can reduce the immune response in chicks caused by *Salmonella* contamination in eggs. In summary, disinfecting *S.* Typhimurium-contaminated chicken eggs with phages may indirectly influence chicks after hatching by reducing *Salmonella* content on eggshells or in the environment, thereby alleviating immune organ damage caused by *Salmonella* contamination in hatched chicks.

Gut microbes play a crucial role in promoting the health and productivity of chicks [44,45,46]. They affect chick growth and are seen as important for predicting future poultry health [47]. However, how gut microbes develop in newly hatched chicks is not fully clear yet [48]. Some studies suggest that the chick’s gut is sterile at birth, and its microbial community mainly depends on the environment it hatches into [49]. The few microbes that chicks initially get largely come from their surroundings, such as the incubator [50]. The eggshell and incubator are the first things chicks encounter after hatching, and these environmental microbes have a big impact on the chick’s gut bacteria. In this study, compared to the positive control group, the phage group showed a significant decrease in abundance in the gut microbiota. This suggests that reducing *Salmonella* on eggshells and in the hatching box environment after phage treatment may help establish a healthier gut microbiota in chicks.

## 4. Materials and Methods

### 4.1. Bacteria Strains

Forty-seven bacterial strains isolated from different provinces of China were used in this study. Detailed information on the bacterial strains is listed in Table 1. Briefly, seven *Salmonella* strains, twenty-nine *Escherichia coli* strains, two *Proteus mirabilis* strains, and two *Klebsiella pneumoniae* strains were isolated from diarrhea–avian sources in Guangxi, respectively. Three *Escherichia coli* strains were isolated from swine sources in Guangdong. One *Escherichia coli* strain and a *Pseudomonas aeruginosa* strain were isolated from avian sources and cat sources in Sichuan. In addition, *S*. Typhimurium CVCC3384, *S*. Enteritidis CVCC1806, *Escherichia coli* CVCC1527, and *Escherichia coli* O157:H7 CVCC 4050 were purchased from the China Veterinary Culture Collection Center (CVCC), Beijing. *S*. Typhimurium SM022 was obtained from the Shanghai Veterinary Research Institute. All the bacterial isolates were used in a spot lysis assay for host range. They were kept at −80 °C in 30% (*v*/*v*) glycerol, and these strains were kept at the clinical veterinary laboratory of Guangxi University. In addition, serotyping of the above strains was performed using a slide-agglutination test using Diagnostic Sera for Entero pathogenic *Escherichia coli* and *Salmonella* (TR301 × 15 and TR102 × 30, Ningbo Tianrun Biopharmaceutical Co., Ltd., Ningbo, China) according to the manufacturer’s instructions.

### 4.2. Isolation and Concentration of the Phage

After three centrifugations (3000× *g*, 10 min), the sewage sample was filtered through a 0.22-μm Polyethersulfone (PES) Millipore filter to remove the bacteria and phytoplankton. The phages in the sample were then separated using the double-agar layer method [51]. In summary, 100 µL filtered sewage samples were mixed with 100 µL logarithmic growth phase bacteria. The mixture was then injected into 3 mL of semisolid medium melted at 50 °C and poured onto the surface of the solid medium. After culturing the agar plate in an incubator at 37 °C for 24 h, the formation of plaques was observed. If there were plaques on the plate, a plaque was picked out and placed in 1 mL of SM buffer (100 mM NaCl, 8 mM MgSO_4_. 7 H_2_O, 50 mM Tris-HCl, pH = 7.5) and then filtered through a 0.22 μm PES Millipore filter. After the filtrate was gradually diluted, the above procedure was repeated. The infection step was repeated at least three times to ensure that the phage solution was completely purified. The purified phage was stored in the SM buffer at 4 °C.

### 4.3. Determination of Host Range of Phage SP02

Bacterial strains listed in Table 1 were used for host range. The host range of isolated phage SP02 was determined using the spot test [51]. In brief, suspensions of tested strains (100 μL) were mixed with LB containing 0.6% agar (3 mL) serving as the overlay and LB containing 1.2% agar (10 mL) serving as the bottom layer. Phage lysates (5 μL) were spotted onto a double-layer agar plate containing the lawns of target strains and incubated at 37 °C for 8−10 h. The formation of plaque was visually confirmed. The formation of clear plaques was regarded as the lysis of host bacteria by phages while the absence of clear plaques was considered as having no lysis.

### 4.4. Determination of Host Range of Phage SP02

The phage titer of SP02 was increased to 10^9^ PFU/mL (PFU, Plaque Forming Units) and sent to Beijing Zhong Jing Ke Yi Technology Co., Ltd (Beijing, China) for a transmission electron microscopy analysis. Phosphotungstic acid (2% *w*/*v*) negative staining was used to determine the morphology of phages SP02 [52]. Capsid, tail, and tail fiber dimensions were measured on at least 15 phage particles.

### 4.5. Thermolability Sensitivity and Stability

For optimal multiplicity of infection (MOI), phage suspension (100 uL) and an equal volume of SM022 were mixed at MOIs of 0.001, 0.01, 0.1, 1, 10, and 100, and aliquots were taken after incubation for 5 h. Then, phage titers were measured using the double-layer agar method.

One-step growth experiments were performed using a modification of methods described previously [53]. The phage SP02 were mixed with SM022 (MOI = 1) in a 37 °C warm bath after 15 centrifugations at 5000× *g* for 1 min, the supernatant was discarded and the precipitate washed with LB. Then, the mixture was suspended in a preheated LB broth, followed by incubation at 37 °C. Samples were taken at 10 min intervals up to 90 min and immediately diluted, and then phage titers were determined using the method mentioned above. The calculation formula of burst size was as follows: Burst size = phage titer at the end of lysis/number of host strain cells at the beginning of infection. All tests were repeated three times.

### 4.6. Phage DNA Extraction

Phage DNA was extracted using previous methods [54]. In brief, 600 μL of purified phage SP02 (10^9^ PFU/mL) was treated with DNase I (CW20905, CWBIO, Taizhou China) and RNase A (CW0601, CWBIO) to a final concentration of 1 μg/mL. The mixture was incubated at 37 °C for 30 min and then at 65 °C for 10 min to inactivate DNase I. Final concentrations of 0.5% EDTA, 20 ng/mL proteinase K (CW2584M, CWBIO), and 10% SDS (sodium dodecyl sulfate) were added to the mixture and incubated at 56 °C for 1 h. DNA was extracted by adding an equal volume of phenol–chloroform. After a mild oscillation for 1 min, the mixture was centrifuged at 12,000× *g* for 10 min. The aqueous layer was moved to a new tube containing 400 μL of isopropanol (which precipitated DNA) then inverted 6–8 times for mixing. Finally, the DNA extraction kit (AP-MN-BF-VNA-50, Axygen, Wuhan China) extracted phage DNA. The pellet was dissolved in 30 µL of buffer Tris and EDTA (TE), and the isolated nucleic acids were separated using 0.8% agarose gel electrophoresis, stained with ethidium bromide, and analyzed under ultraviolet (UV) light. Purified DNA samples were stored at −80 °C for further use.

### 4.7. Phage Genome Sequencing and Analysis

The method of sequencing adopted was whole genome shotgun (WGS). The sequencing platform was Illumina Miseq (San Diego, CA, USA); sequencing mode was paired-end (2 × 250 bp) and had a library insert size of 400 bp. The complete genome sequence was annotated using Subsystem Technology (RAST, Ver: 1.3.0, http://rast.nmpdr.org, accessed on 21 September 2023) and GeneMark, Ver: 4.30, http://opal.biology.gatech.edu/GeneMark/, accessed on 21 September 2023) [55,56]. All predicted open reading frames (ORFs) were verified using the online BLASTP (Ver: 2.16.0, http://www.ncbi.nlm.nih.gov/BLAST, accessed on 21 September 2023). The putative transfer RNA (tRNA)-encoding genes were searched using tRNA scan-SE (Ver: 2.012, http://trna.ucsc.edu/tRNAscan-SE/, accessed on 21 September 2023) [57]. Putative virulence factors and antimicrobial resistance genes were screened using the Virulence Factor Database [58] and the Comprehensive Antibiotic Resistance Database [59], respectively. A comparative circular genome map of the phage SP02 genome was created using CG View Server [60] and linear comparison figures of multiple genomic loci were created using Easy-fig_2.2.5_win based on BLAST [61]. The phylogenetic tree was generated in MEGA 7 using the neighbor-joining method with *p* distance values and bootstrap replicates of 1000 [62].

### 4.8. Protective Effect of Aerosol Spraying of Phage on Salmonella-Contaminated Chicken Embryos

The 135 White Leghorn eggs were purchased from the SPF Experimental Animal Center of Da Huanong Poultry & Egg Co. (Guangzhou, China). A total of 5% Neosporin at 1:1000 was mixed into a disinfectant solution and sprayed onto the 1-day-old eggs surface and dried. Eggs of similar weights were placed in the incubator, and incubation conditions followed the preset procedures for the incubator. A total of 135 eggs with normal growth were randomly selected and divided into 3 groups, with 45 eggs in each parallel group. The negative group was sprayed with sterilized water. The phage group and the without phage group were challenged with 100 mL of 10^9^ CFU/mL of *S*. Typhimurium SM022 by aerosol spray. Immediately afterward, the phage group was treated with 50 mL of phage suspension of 10^9^ PFU/mL by aerosol spray. The other two groups were treated with the same volume of SM buffer. The *Salmonella* culture, phage suspension, and sterilized water were applied with a manual spray, delivering approximately 0.5 mL/s. The three groups were placed in separate incubators.

After hatching, all chicks were placed in brooder rings with free feed and water and maintained at an appropriate temperature and humidity for 14 days. During this time, the chicks were examined for limping (arthritis) and accumulation of feces around the cloacae (pasting). At 1, 7, and 14 days, three chicks were humanely euthanized with isoflurane to obtain samples of liver, spleen, jejunum, and ceca, harvested for bacterial counts. Sterilely collected tissue of every group was weighed and 0.1 g was homogenized by grinding it in 900 μL of saline buffer. After diluting the homogenate 10 times, 100 μL of the homogenate was coated in bismuth sulfite agar and incubated at 37 °C for 18−24 h. Bacterial counts of one sample were repeated three times. Frozen sections of aseptically collected jejunum were observed using a laser confocal microscope for fluorescence emitted by *Salmonella* SM022. Cecum content samples were collected in 1.5–2 mL sterile polypropylene tubes and were immediately frozen in liquid nitrogen, and then kept at −80 °C until microbiome analysis. The liver, thymus, spleen, and bursa were stripped of fat, weighed separately, and the organ indices were calculated.

### 4.9. 16S rRNA Amplicon Sequencing and Bioinformatic Analyses

The 16S amplicon sequencing and analysis were conducted by OE biotech Co., Ltd. (Shanghai, China). Total genomic DNA was extracted using DNA Extraction Kit (CW2298S, CWBIO, Jiangsu China) following the manufacturer’s instructions. Quality and quantity of DNA was verified with NanoDrop (Thermo Fisher Waltham, MA, USA) and agarose gel. Extracted DNA was diluted to a concentration of 1 ng/μL and stored at −20 °C until further processing. The diluted DNA was used as a template for PCR amplification of bacterial 16S rRNA genes with the barcoded primers and Takara Ex Taq (RR001Q, Takara, Osaka, Japan). For bacterial diversity analysis, V3-V4 variable regions of 16S rRNA genes were amplified with universal primers 343F (5′-TACGGRAGGCAGCAG-3′) and 798R (5′-AGGGTATCTAATCCT-3′). Amplicon quality was visualized using electrophoresis, purified with AMPure XP beads (Agencourt, Beckman Coulter, Brea, CA, USA), and amplified for another round of PCR. After purification with the AMPure XP beads again, the final amplicons were quantified using a Qubit dsDNA assay kit (Q32850, Invitrogen, Waltham, MA, USA). Equal amounts of purified amplicon were poled for subsequent sequencing.

Bioinformatics analysis methods were referenced from Callahan [63]. Raw sequencing data were in FASTQ format. Paired-end reads were then preprocessed using cutadapt software (Ver: 4.9) to detect and cut off the adapter. After, paired-end reads were filtered to remove low-quality sequences, denoised, merged, and the chimera rads were removed using DADA2 with the default parameters of the QIME2 (02.1) [64]. Finally, the software output the representative rads and the ASV abundance table. The representative read of each ASV was selected using the QIME2 package. All representative rads were annotated and blasted against Silva database Version 138 (16s rDNA, https://www.arb-silva.de/documentation/release-138/, accessed on 21 September 2023) using a q2 feature classifier with the default parameters.

### 4.10. Statistical Analysis

Statistical analyses were performed with GraphPad Prism 8.0.2 (GraphPad Software, Inc., San Diego, CA, USA). The significance of the experimental data was determined using multiple *t*-tests. The error line represents the standard deviation (SD) of the mean. All the experiments were conducted in triplicate.

## 5. Conclusions

Our study demonstrated that the application of a phage in removing *Salmonella* from the egg surface during the process of incubating eggs showed good functionality. The results showed that phage SP02 possessed good in vitro bacterial inhibitory effects, and spraying the phage on the eggshell surface could reduce the effect of *Salmonella* on the hatching rate and further improve the health of young chicks. In this research, it is believed that phages have certain application prospects as an egg disinfectant, which provides the theoretical basis and experimental foundation for the next application of phages.

## Figures and Tables

**Table 1:** Bacterial strain information.

Strains	Genus	Place	Source	Serotype/Capsular Type	Sensitivity
GXSE-S7	*Salmonella*	Guangxi	Pig	O2, -: Ha: -	−
GXSE-S4	*Salmonella*	Guangxi	Pig	O7, -: Hc: -	−
CVCC3384	*Salmonella*	CVCC	Pig	*S.* Typhimurium	+
CVCC1806	*Salmonella*	CVCC	Avian	*S.* Enteritidis	−
SF-0923	*Salmonella*	Fujian	Avian	*S.* Pullorum	+
SX-1014	*Salmonella*	Jiangsu	Avian	*S.* Pullorum	+
SM022	*Salmonella*	Shanghai	-	*S.* Typhimurium	+
CVCC1527	*Escherichia coli*	CVCC	Pig	O8: K88	−
CVCC4050	*Escherichia coli*	CVCC	-	O157: H7	−
GXEC-1	*Escherichia coli*	Guangxi	Pig	undetected	−
GXEC-2	*Escherichia coli*	Guangxi	Pig	undetected	−
GXEC-3	*Escherichia coli*	Guangxi	Pig	undetected	−
GXEC-4-1	*Escherichia coli*	Guangxi	Pig	undetected	−
GXEC-6	*Escherichia coli*	Guangxi	Pig	undetected	−
GXEC-7-1	*Escherichia coli*	Guangxi	Pig	undetected	−
GXEC-8	*Escherichia coli*	Guangxi	Pig	undetected	−
GXEC-9	*Escherichia coli*	Guangxi	Pig	undetected	−
GXEC-10	*Escherichia coli*	Guangxi	Pig	undetected	−
GXEC-11-1	*Escherichia coli*	Guangxi	Pig	undetected	−
GXEC-11-2	*Escherichia coli*	Guangxi	Pig	undetected	−
GXEC-12	*Escherichia coli*	Guangxi	Pig	undetected	−
GXEC-13	*Escherichia coli*	Guangxi	Pig	undetected	−
GXEC-H6	*Escherichia coli*	Guangxi	Pig	O86: K61	−
GXEC-I11	*Escherichia coli*	Guangxi	Pig	undetected	−
GXEC-K5	*Escherichia coli*	Guangxi	Pig	O114: K90	−
GXEC-H1	*Escherichia coli*	Guangxi	Pig	undetected	−
GXEC-H2- 1	*Escherichia coli*	Guangxi	Pig	undetected	−
GXEC-H2- 2	*Escherichia coli*	Guangxi	Pig	undetected	−
GXEC-H3	*Escherichia coli*	Guangxi	Pig	undetected	−
GXEC-H4	*Escherichia coli*	Guangxi	Pig	undetected	−
GXEC-H7	*Escherichia coli*	Guangxi	Pig	undetected	−
GXEC-Z1-1	*Escherichia coli*	Guangxi	Pig	undetected	−
GXEC-Z1-2	*Escherichia coli*	Guangxi	Pig	undetected	−
GXEC-Z1-3	*Escherichia coli*	Guangxi	Pig	undetected	−
GXEC-Z2-1	*Escherichia coli*	Guangxi	Pig	undetected	−
GXEC-Z2-2	*Escherichia coli*	Guangxi	Pig	undetected	−
GXEC-E11	*Escherichia coli*	Guangxi	Pig	undetected	−
GXEC-E4-3	*Escherichia coli*	Guangxi	Environment	undetected	−
SCEC-E19	*Escherichia coli*	Sichuan	Avian	undetected	−
GDEC-E3	*Escherichia coli*	Guangdong	Avian	O127: K63	−
GDEC-E6-1	*Escherichia coli*	Guangdong	Pig	undetected	−
GDEC-E6-2	*Escherichia coli*	Guangdong	Pig	undetected	−
GXPM-05	*Proteus mirabilis*	Guangxi	Pig	undetected	−
GXPM-08	*Proteus mirabilis*	Guangxi	Pig	undetected	−
GXKP-J05	*Klebsiella pneumoniae*	Guangxi	Pig	K20	−
GXKP-JM2	*Klebsiella pneumoniae*	Guangxi	Pig	undetected	−
SZPA-PA1	*Pseudomonas aeruginosa*	Shenzhen	Cat	undetected	+

“+” symbolizes positive, strain was lysed; “−” symbolizes negative, strains were not lysed. Undetected means the strain did not belong to these serotypes (O157:H7, O157, O114:K90, O126:K71, O26:K60, O142:K86, O127a: K63, and O111:K58) or these capsular types (K1, K2, K5, K20, K54, and K57).

## Data Availability

The annotated complete genome sequence of the phage SP02 was deposited in GenBank under the access number OM967031.

## References

[1] Toro H., Price S.B., McKee A.S., Hoerr F.J., Krehling J., Perdue M., Bauermeister L. (2005). Use of bacteriophages in combination with competitive exclusion to reduce *Salmonella* from infected chickens. Avian. Dis..

[2] Kumar Y., Singh V., Kumar G., Gupta N.K., Tahlan A.K. (2019). Serovar diversity of *Salmonella* among poultry. Indian J. Med. Res..

[3] Chousalkar K., Gole V., Caraguel C., Rault J.L. (2016). Chasing *Salmonella* Typhimurium in free range egg production system. Vet. Microbiol..

[4] Hiley L., Graham R.M.A., Jennison A.V. (2019). Genetic characterisation of variants of the virulence plasmid, pSLT, in *Salmonella* enterica serovar Typhimurium provides evidence of a variety of evolutionary directions consistent with vertical rather than horizontal transmission. PLoS ONE.

[5] Scott T.A., Swetnam C. (1993). Screening Sanitizing Agents and Methods of Application for Hatching Eggs II. Effectiveness Against Microorganisms on the Egg Shell. J. Appl. Poult. Res..

[6] Batkowska J., Al-Shammari K.I.A., Lukasz W., Nowakowicz-Debek B., Gryzinska M. (2018). Evaluation of propolis extract as a disinfectant of Japanese quail (*Coturnix coturnix japonica*) hatching eggs. Poult. Sci..

[7] Olsen R., Kudirkiene E., Thofner I., Pors S., Karlskov-Mortensen P., Li L., Papasolomontos S., Angastiniotou C., Christensen J. (2017). Impact of egg disinfection of hatching eggs on the eggshell microbiome and bacterial load. Poult. Sci..

[8] Oliveira G.d.S., McManus C., Salgado C.B., dos Santos V.M. (2022). Effects of sanitizers on microbiological control of hatching eggshells and poultry health during embryogenesis and early stages after hatching in the last decade. Animals.

[9] De Faria F.A., Filho G.D.M.O., Neves J., de Siqueira P.S., de Oliveira L.F., de Oliveira I.P. (2014). Incubatorios-controle de qualidade [Hatcheries–quality control]. Rev. Eletrônica Fac. Montes. Belos..

[10] Zeweil H.S., Rizk R.E., Bekhet G.M., Ahmed M.R. (2015). Comparing the effectiveness of egg disinfectants against bacteria and mitotic indices of developing chick embryos. J. Basic Appl. Zool..

[11] Kusstatscher P., Cernava T., Liebminger S., Berg G. (2017). Replacing conventional decontamination of hatching eggs with a natural defense strategy based on antimicrobial, volatile pyrazines. Sci. Rep..

[12] Debes A., Basyony M. (2011). The use of oregano (*Origanum vulgare* L.) and ginger (*Zingiber officinale*) oils as alternative hatching egg disinfectant versus formaldhyde fumigation in leghorn and matrouh eggs. Ind. Crops Prod..

[13] Wells J.B., Coufal C.D., Parker H.M., Kiess A.S., Mcdaniel C.D. (2011). Hatchablility of Broiler Breeder Eggs Sanitized with a Combination of Ultraviolet Light and Hydrogen Peroxide*. Int. J. Poult. Sci..

[14] Pees M., Motola G., Hafez M.H., Bachmeier J., Bruggemann-Schwarze S., Tebrun W. (2020). Use of electron irradiation versus formaldehyde fumigation as hatching egg disinfectants—Efficacy and impact on hatchability and broiler performance. Tierarztl. Prax. Ausg. G Grosstiere Nutztiere.

[15] Sander J.E., Wilson J.L. (1999). Effect of hydrogen peroxide disinfection during incubation of chicken eggs on microbial levels and productivity. Avian. Dis..

[16] Maharjan P., Cox S., Gadde U., Clark F.D., Bramwell K., Watkins S.E. (2017). Evaluation of chlorine dioxide based product as a hatchery sanitizer. Poult. Sci..

[17] Wlazlo L., Drabik K., Al-Shammari K.I.A., Batkowska J., Nowakowicz-Debek B., Gryzinska M. (2020). Use of reactive oxygen species (ozone, hydrogen peroxide) for disinfection of hatching eggs. Poult. Sci..

[18] Melo L.D.R., Oliveira H., Pires D.P., Dabrowska K., Azeredo J. (2020). Phage therapy efficacy: A review of the last 10 years of preclinical studies. Crit. Rev. Microbiol..

[19] Kortright K.E., Chan B.K., Koff J.L., Turner P.E. (2019). Phage Therapy: A Renewed Approach to Combat Antibiotic-Resistant Bacteria. Cell Host Microbe.

[20] Merikanto I., Laakso J.T., Kaitala V. (2018). Outside-host phage therapy as a biological control against environmental infectious diseases. Theor. Biol. Med. Model.

[21] Donlan R.M. (2009). Preventing biofilms of clinically relevant organisms using bacteriophage. Trends Microbiol..

[22] Doss J., Culbertson K., Hahn D., Camacho J., Barekzi N. (2017). A Review of Phage Therapy against Bacterial Pathogens of Aquatic and Terrestrial Organisms. Viruses.

[23] Zaczek M., Weber-Dabrowska B., Gorski A. (2020). Phages as a Cohesive Prophylactic and Therapeutic Approach in Aquaculture Systems. Antibiotics.

[24] Kowalska J.D., Kazimierczak J., Sowinska P.M., Wojcik E.A., Siwicki A.K., Dastych J. (2020). Growing Trend of Fighting Infections in Aquaculture Environment—Opportunities and Challenges of Phage Therapy. Antibiotics.

[25] Endersen L., O’Mahony J., Hill C., Ross R.P., McAuliffe O., Coffey A. (2014). Phage therapy in the food industry. Annu. Rev. Food Sci. Technol..

[26] Pires D.P., Costa A.R., Pinto G., Meneses L., Azeredo J. (2020). Current challenges and future opportunities of phage therapy. FEMS Microbiol. Rev..

[27] Motlagh A.M., Bhattacharjee A.S., Goel R. (2016). Biofilm control with natural and genetically-modified phages. World J. Microbiol. Biotechnol..

[28] Chan B.K., Sistrom M., Wertz J.E., Kortright K.E., Narayan D., Turner P.E. (2016). Phage selection restores antibiotic sensitivity in MDR Pseudomonas aeruginosa. Sci. Rep..

[29] Samson J.E., Magadan A.H., Sabri M., Moineau S. (2013). Revenge of the phages: Defeating bacterial defences. Nat. Rev. Microbiol..

[30] Gantois I., Ducatelle R., Pasmans F., Haesebrouck F., Gast R., Humphrey T.J., Van Immerseel F. (2009). Mechanisms of egg contamination by *Salmonella enteritidis*. FEMS Microbiol. Rev..

[31] Luong T., Salabarria A.C., Edwards R.A., Roach D.R. (2020). Standardized bacteriophage purification for personalized phage therapy. Nat. Protoc..

[32] Khan S., McWhorter A.R., Moyle T.S., Chousalkar K.K. (2021). Refrigeration of eggs influences the virulence of Salmonella Typhimurium. Sci. Rep..

[33] Bernardini L., Barbosa E., Charao M.F., Brucker N. (2022). Formaldehyde toxicity reports from *in vitro* and *in vivo* studies: A review and updated data. Drug Chem. Toxicol..

[34] Schoeni J.L., Glass K.A., McDermott J.L., Wong A.C. (1995). Growth and penetration of Salmonella enteritidis, Salmonella heidelberg and Salmonella typhimurium in eggs. Int. J. Food Microbiol..

[35] S S.K., Bhat S.G. (2021). In vitro efficiency evaluation of phage cocktail for biocontrol of *Salmonella* spp. in food products. Arch. Microbiol..

[36] Huang J., Liang L., Cui K., Li P., Hao G., Sun S. (2022). Salmonella phage CKT1 significantly relieves the body weight loss of chicks by normalizing the abnormal intestinal microbiome caused by hypervirulent Salmonella Pullorum. Poult. Sci..

[37] Waseh S., Hanifi-Moghaddam P., Coleman R., Masotti M., Ryan S., Foss M., MacKenzie R., Henry M., Szymanski C.M., Tanha J. (2010). Orally administered P22 phage tailspike protein reduces salmonella colonization in chickens: Prospects of a novel therapy against bacterial infections. PLoS ONE.

[38] Bardina C., Spricigo D.A., Cortes P., Llagostera M. (2012). Significance of the bacteriophage treatment schedule in reducing Salmonella colonization of poultry. Appl. Environ. Microbiol..

[39] Senevirathne A., Hewawaduge C., Lee J.H. (2021). Immunization of chicken with flagellin adjuvanted Salmonella enteritidis bacterial ghosts confers complete protection against chicken salmonellosis. Poult. Sci..

[40] Glick B. (1978). The immune response in the chicken: Lymphoid development of the bursa of Fabricius and thymus and an immune response role for the gland of Harder. Poult. Sci..

[41] Ali O.H., Elzubeir E.A., Elhadi H.M. (2008). Effect of season on the immunity of newly hatched broiler chicks reared in arid-hot climate. Pak. J. Biol. Sci..

[42] Awaya K., Tomonaga S., Sakai K., Tashiro J. (1975). Lymphoid nodules in the thymus of the chicken. Okajimas Folia Anat. Jpn..

[43] Chen C., Li J., Zhang H., Xie Y., Xiong L., Liu H., Wang F. (2020). Effects of a probiotic on the growth performance, intestinal flora, and immune function of chicks infected with Salmonella pullorum. Poult. Sci..

[44] Abdelwhab E.M., Grund C., Aly M.M., Beer M., Harder T.C., Hafez H.M. (2012). Influence of maternal immunity on vaccine efficacy and susceptibility of one day old chicks against Egyptian highly pathogenic avian influenza H5N1. Vet. Microbiol..

[45] Waite D.W., Taylor M.W. (2015). Exploring the avian gut microbiota: Current trends and future directions. Front. Microbiol..

[46] Zhuang L., Chen H., Zhang S., Zhuang J., Li Q., Feng Z. (2019). Intestinal Microbiota in Early Life and Its Implications on Childhood Health. Genom. Proteom. Bioinform..

[47] Magrath R.D. (1991). Nestling weight and juvenile survival in the blackbird, Turdus merula. J. Anim. Ecol..

[48] Bare L.N., Wiseman R.F. (1964). Delayed Appearance of Lactobacilli in the Intestines of Chicks Reared in a “New” Environment. Appl. Microbiol..

[49] Huang T., Han J., Liu Y., Fei M., Du X., He K., Zhao A. (2023). Dynamic distribution of gut microbiota in posthatching chicks and its relationship with average daily gain. Poult. Sci..

[50] Ding J., Dai R., Yang L., He C., Xu K., Liu S., Zhao W., Xiao L., Luo L., Zhang Y. (2017). Inheritance and Establishment of Gut Microbiota in Chickens. Front. Microbiol..

[51] Han J.E., Kim J.H., Hwang S.Y., Choresca C.H., Shin S.P., Jun J.W., Chai J.Y., Park Y.H., Park S.C. (2013). Isolation and characterization of a Myoviridae bacteriophage against Staphylococcus aureus isolated from dairy cows with mastitis. Res. Vet. Sci..

[52] Ackermann H.-W., Clokie M.R.J., Kropinski A.M. (2009). Basic Phage Electron Microscopy. Bacteriophages: Methods and Protocols, Volume 1: Isolation, Characterization, and Interactions.

[53] Shende R.K., Hirpurkar S.D., Sannat C., Rawat N., Pandey V. (2017). Isolation and characterization of bacteriophages with lytic activity against common bacterial pathogens. Vet. World.

[54] Zhang C., Yuan J., Guo C., Ge C., Wang X., Wei D., Li X., Si H., Hu C. (2021). Identification and complete genome of lytic “Kp34likevirus” phage vB_KpnP_Bp5 and therapeutic potency in the treatment of lethal *Klebsiella pneumoniae* infections in mice. Virus Res..

[55] Aziz R.K., Bartels D., Best A.A., DeJongh M., Disz T., Edwards R.A., Formsma K., Gerdes S., Glass E.M., Kubal M. (2008). The RAST Server: Rapid annotations using subsystems technology. BMC Genom..

[56] Besemer J., Borodovsky M. (2005). GeneMark: Web software for gene finding in prokaryotes, eukaryotes and viruses. Nucleic Acids Res..

[57] Lowe T.M., Eddy S.R. (1997). tRNAscan-SE: A program for improved detection of transfer RNA genes in genomic sequence. Nucleic Acids Res..

[58] Liu B., Zheng D., Jin Q., Chen L., Yang J. (2019). VFDB 2019: A comparative pathogenomic platform with an interactive web interface. Nucleic Acids Res..

[59] Jia B., Raphenya A.R., Alcock B., Waglechner N., Guo P., Tsang K.K., Lago B.A., Dave B.M., Pereira S., Sharma A.N. (2017). CARD 2017: Expansion and model-centric curation of the comprehensive antibiotic resistance database. Nucleic Acids Res..

[60] Grant J.R., Stothard P. (2008). The CGView Server: A comparative genomics tool for circular genomes. Nucleic Acids Res..

[61] Sullivan M.J., Petty N.K., Beatson S.A. (2011). Easyfig: A genome comparison visualizer. Bioinformatics.

[62] Kumar S., Stecher G., Li M., Knyaz C., Tamura K. (2018). MEGA X: Molecular Evolutionary Genetics Analysis across Computing Platforms. Mol. Biol. Evol..

[63] Callahan B.J., McMurdie P.J., Rosen M.J., Han A.W., Johnson A.J., Holmes S.P. (2016). DADA2: High-resolution sample inference from Illumina amplicon data. Nat. Methods.

[64] Bolyen E., Rideout J.R., Dillon M.R., Bokulich N.A., Abnet C.C., Al-Ghalith G.A., Alexander H., Alm E.J., Arumugam M., Asnicar F. (2019). Reproducible, interactive, scalable and extensible microbiome data science using QIIME 2. Nat. Biotechnol..

